# Highly sensitive and fast-response ethanol sensing of porous Co_3_O_4_ hollow polyhedra *via* palladium reined spillover effect†

**DOI:** 10.1039/d1ra09352e

**Published:** 2022-02-28

**Authors:** Guotao Yuan, Yihong Zhong, Yufeng Chen, Qiqi Zhuo, Xuhui Sun

**Affiliations:** Institute of Functional Nano and Soft Materials Laboratory (FUNSOM), Soochow University Suzhou 215123 China zqq88263268@126.com xhsun@suda.edu.cn; College of Material Science & Engineering, Jiangsu University of Science and Technology Zhenjiang China

## Abstract

Highly sensitive and fast detection of volatile organic compounds (VOCs) in industrial and living environments is an urgent need. The combination of distinctive structure and noble metal modification is an important strategy to achieve high-performance gas sensing materials. In addition, it is urgent to clarify the chemical state and function of noble metals on the surface of the sensing material during the actual sensing process. In this work, Pd modified Co_3_O_4_ hollow polyhedral (Pd/Co_3_O_4_ HP) is developed through one-step pyrolysis of a Pd doped MOF precursor. At an operating temperature of 150 °C, the Pd/Co_3_O_4_ HP gas sensor can achieve 1.6 times higher sensitivity than that of Co_3_O_4_ HP along with fast response (12 s) and recovery speed (25 s) for 100 ppm ethanol vapor. Near-ambient pressure X-ray photoelectron spectroscopy (NAPXPS) was used to monitor the dynamic changes in the surface state of Pd/Co_3_O_4_ HP. The NAPXPS results reveal that the oxidation and reduction of Pd in the ethanol sensing process are attributed to a spillover effect of oxygen and ethanol, respectively. This work opens up an effective approach to investigate spillover effects in a sensing mechanism of noble metal modified oxide semiconductor sensors.

## Abstract 2

Pd/Co_3_O_4_ HP was developed by simple pyrolysis of Pd doped MOF, which achieved high sensitivity with fast response (12 s)/recovery speed (25 s) for 100 ppm ethanol. APXPS results provide experimental evidence to enhance performance by Pd spillover effect.

## Graphical abstract

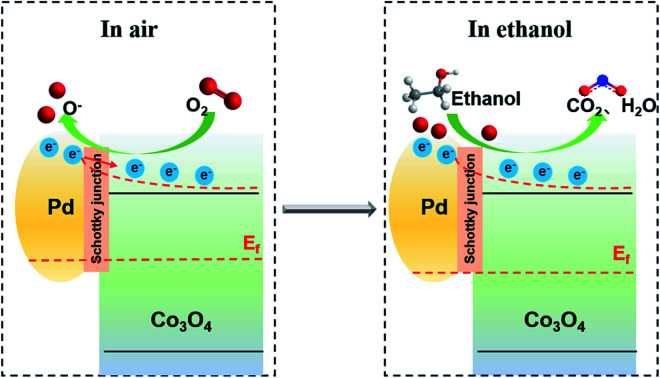

## 1. Introduction

Monitoring of volatile organic compounds (VOCs) is becoming ever more important due to stringent environmental regulations and increasing health concerns.^1–3^ Nowadays, many efforts have been made to improve sensor performance, such as developing high-performance sensing materials and optimizing the sensor structure. Metal oxide semiconductors (MOS) are some of the candidate materials for detection of gas concentration due to their high sensitivity, low cost, good stability and so on.^4,5^ However, their high operating temperature and still unsatisfactory response/recovery speed hinder their further applications.^6^

In general, porous structured materials are beneficial to gas sensing performance by increasing the surface reactive sites and facilitating the diffusion of target gases.^7–9^ For example, Chen *et al.*^8^ fabricated a mesoporous Co_3_O_4_ nanosheet array supported on an N-doped carbon foam for enhanced ethanol sensing application. The working temperature can be decreased to 100 °C and the limit of detection lowers to 0.2 ppm. Metal organic framework (MOF)-derived MOS sensor can provide the less agglomerated nanostructures and larger active site of the sensing materials to obtain higher sensing performance. For examples, Masuda *et al.*^10^ successfully synthesized highly porous CuO nanostructures by converting Cu-MOF precursor, which could detect a tiny concentration of acetone gas up to 50 ppb. Zhang *et al.*^11^ fabricated porous hollow Co_3_O_4_@ZnO cages through ZIF-67@ZIF-8-derived process, which exhibited a high sensitivity of ∼41 with a response/recovery time of only 3/2 s for 33 ppm trimethylamine.

As an additive with excellent catalytic ability, Pd, Pt or Au are good auxiliary materials, in which the electronic sensitization and spillover effect (chemical sensitization) can significantly promote the gas sensing performance. Chen *et al.*^12^ fabricated interfacial heterostructures in Pd_7.18%_W_18_O_49_ nanowires by doping different Pd contents, which showed the best sensing response and the fastest response–recovery speeds (5 and 10 s, respectively) at a working temperature of 175 °C. Umarji *et al.*^13^ reported a Cr, Pt co-doping SnO_2_ thin film sensor with enhanced selectivity due to specific catalytic effect and synergistic effect. Moreover, AuPd alloys modified SnO_2_ sensors were fabricated by Duan *et al.* and exhibited high response value and selectivity to dimethyl disulfide. The enhanced performance was due to sulfur spillover driven by charge transfer between AuPd alloys and SnO_2_.^14^

Despite these great efforts, noble metal-drived spillover effect often is used to clarify the sensing mechanism in metal–support oxide sensors.^15,16^ Many techniques have been applied to study the noble metal-drived spillover effect in metal–support oxide sensors, including X-ray photoelectron spectroscopy (XPS) and ultraviolet photoelectron spectroscopy (UPS). However, limited to ultrahigh vacuum conditions, it's difficult to identify the surface core-level spectra in the atomic scale, which is beneficial for the clarification of the spillover effect. Near ambient-pressure X-ray photoelectron spectroscopy (NAPXPS) can measure the core level spectra of materials and gas molecules *in situ* under near ambient pressure conditions. Recently, NAPXPS has been successfully used for the investigation of spillover effect in catalytic reaction.^17–19^ For instance, Somorjai *et al.*^17^ used NAPXPS technique to monitoring Ce 4d core level and found cerium oxide was reduced at lower temperatures for Pt/CeO_2_ catalyst than for pure mesoporous CeO_2_. This result identified the spillover effect of atomic hydrogen from Pt to ceria surface. Therefore, further investigation of spillover effect in gas sensor by NAPXPS is still necessary.

In this work, Pd modified Co_3_O_4_ hollow polyhedral with a porous structure through one-step pyrolysis of Pd-ZIF-67 composites was synthesized and fabricated as a gas sensor. The as-prepared sensor exhibited high responsibility (∼20.8) and fast response/recovery speed (12 s/25 s) for the selective detection of 100 ppm ethanol vapor at an operating temperature of 150 °C. NAPXPS was used to monitor the dynamic changes in the surface state of Pd/Co_3_O_4_ HP to further elucidate the sensing mechanism.

## 2. Experiments

### Materials

Palladium chloride (PdCl_2_, 98%), cobalt(ii) nitrate hexahydrate (Co(NO_3_)_2_·6H_2_O, 98.5%), 2-methylimidazole (C_4_H_6_N_2_, 2MeIm, 99.0%), hydrochloric acid (HCl, 30%) and methanol (CH_5_OH, 99.5%) were purchased from Sinopharm Chemical Reagent Co. Ltd (Shanghai, China).

### Synthesis of Pd-ZIF-67

Firstly, 0.5 mL H_2_PdCl_4_ (50 mM) solution and 0.25 g Co(NO_3_)_2_·6H_2_O were dissolved in 50 mL methanol. Then 0.7 g 2-MeIm was dissolved in 50 mL methanol and the former solution was poured in it at 1500 rpm for 5 min. Afterwards the mixed solution was kept at room temperature for 5 h to get the products. Finally, the products were washed with absolute ethanol for three times and dried at 50 °C overnight to obtain Pd-ZIF-67.

### Synthesis of Pd–Co_3_O_4_ HP

Pd/Co_3_O_4_ HP were synthesized through one-step pyrolysis of Pd-ZIF-67 composites in the furnace at 500 °C for 2 h in air.

### Materials characterization

Field-emission scanning electron microscopy (FESEM, Zeiss G500) and transmission electron microscopy (TEM, Thermo Fisher Talos F200X) equipped with energy dispersive X-ray spectroscopy (EDX) were carried out to characterize morphologies and structure of the samples. X-ray diffraction (XRD) patterns were collected on a PANalytical X-ray diffractometer by Cu Kα source.

### Fabrication of Pd/Co_3_O_4_ HP gas sensor

Firstly, the Au interdigital electrodes were deposited on insulating silicon wafer substrate (2 cm × 1 cm). Then, 0.2 mL of Pd/Co_3_O_4_ HP dispersion (0.2 g mL^−1^) was coated on the substrate to fabricate a simple Pd/Co_3_O_4_ HP sensor. Before use, the sensor was aged by thermal treatment at 100 °C for 12 h.

### Gas sensor measurements

The sensing performance of Pd/Co_3_O_4_ HP sensor was assessed by a reported sensing system composed of an electrical test system (Keithley-2400) and a home-made gas distribution system. The gas chamber volume was around 1 L and the bias voltage was set at 1 V. Different atmospheres were pumped to the testing chamber at a gas flow rate of 200 sccm. The sensitivity of gas response was defined as the ratio ((*R*_g_ − *R*_a_)/*R*_a_) of the resistance in air (*R*_a_) to that in the mix gases (*R*_g_). Response and recovery times were defined as the time required for 90% of the total resistance change upon exposure to gas and air, respectively.

### Near ambient pressure X-ray photoelectron spectroscopy (NAPXPS) measurement

The synchrotron-based NAPXPS measurements were operated at TLS 24 A. The incident photon energy was adjusted from 530 eV to 730 eV for detecting the electronic state information of elements with the same atomic layer thickness. Au 4f_7/2_ peak (84 eV) was used for the calibration of the photon energy. Laser irradiation was used to heat the sensors. O 1s and Pd 3d spectrum of Pd/Co_3_O_4_ HP sensor were collected at a pass energy of 20 eV under different atmosphere (ultra-high vacuum (UHV), 1 mbar O_2_, and 1 mbar O_2_/0.2 mbar ethanol mixed gas, respectively). CasaXPS was used for data processing of NAPXPS spectrum.

## 3. Results and discussion

### Synthesis and characterizations

**Fig. 1:**
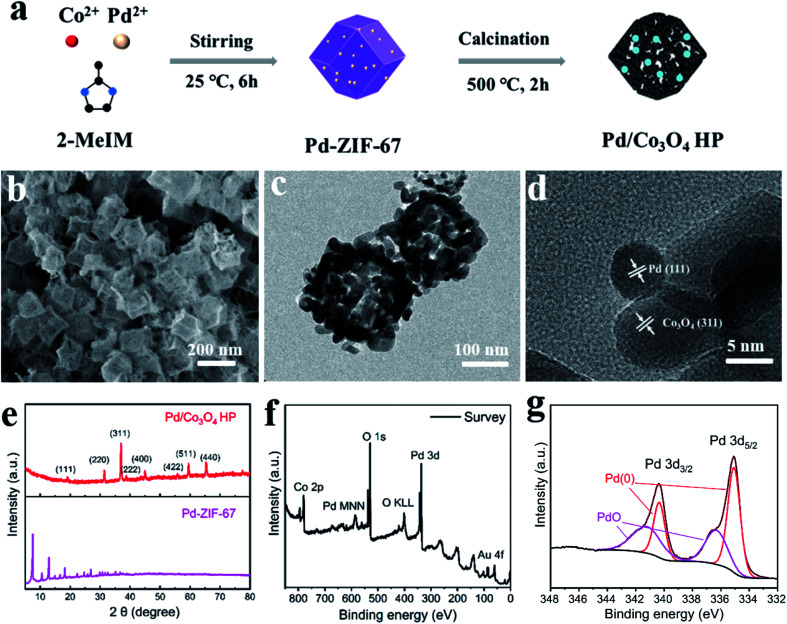
The preparation and characterizations of Pd/Co_3_O_4_ HP. (a) Schematic illustration of the synthetic process; (b) SEM image; (c) TEM image; (d) HRTEM image; (e) XRD patterns; (f) XPS survey and (g) Pd 3d spectra.

Pd modified Co_3_O_4_ hollow polyhedra (Pd/Co_3_O_4_ HP) was synthesized by one-step pyrolysis of Pd-ZIF-67 composites, as shown in Fig. 1a. Pd-ZIF-67 nanocomposites were firstly prepared through a coprecipitation method. SEM images (Fig. S1a and b†) indicate the size of around 200 nm and monodispersion of Pd-ZIF-67 composites.The elemental compositions of Pd-ZIF-67 were characterized by STEM. TEM images (Fig. S1c†) and corresponding EDX mappings (Fig. S1d†) verified the doping of Pd on the surface.

Subsequently, Pd-ZIF-67 were calcinated at 500 °C in air to form Pd/Co_3_O_4_ HP. The SEM image (Fig. 1b) show that as-prepared samples presented porous polyhedron morphology and uniform monodispersion with a size of around 200 nm. The TEM images (Fig. 1c) show that Pd/Co_3_O_4_ hollow polyhedrons are composed of small nanoparticles. In addition, the distinct lattice fringes observed in high-resolution TEM (Fig. 1d) are assigned to Pd (111) plane and Co_3_O_4_ (311) plane. Typical diffraction peaks in XRD patterns (Fig. 1e) can be well assigned to Co_3_O_4_ phase (PDF#42-1467) and ZIF structure, respectively. No Pd peaks were observed in XRD patterns, which could be due to the low content of Pd. XPS was carried out to study the electronic structure of Pd as showed in Fig. 1f and g. The XPS survey verified the composition of Co, O, Pd, C and Au (from Au electrodes) elements. Further fine scanning of Pd 3d and peak fitting results show that there are four characteristic peaks at 335.1 eV, 335.8 eV, 340.4 eV and 341.2 eV. The peaks at 335.1 eV (340.4 eV) and 335.8 eV (341.2 eV) correspond to metallic Pd and PdO, respectively. This result confirms that Pd was successfully modified on the surface of Co_3_O_4_ HP and partially oxidized.

### Sensing performance

**Fig. 2:**
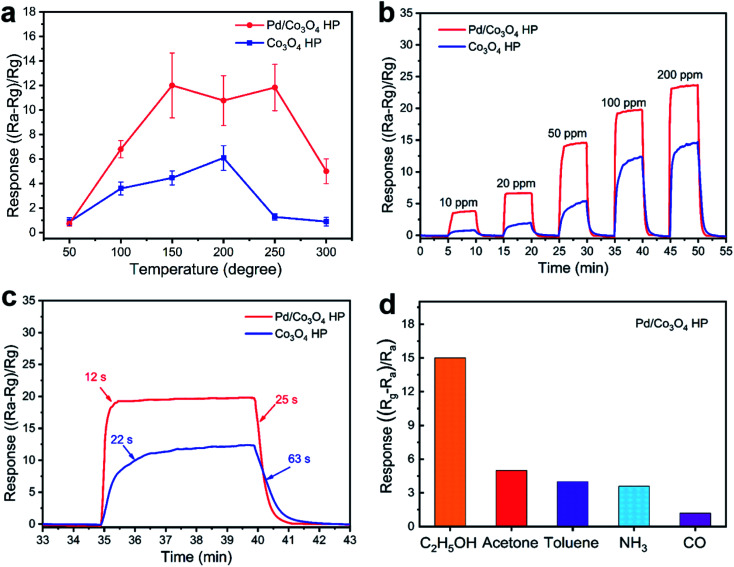
Sensing properties of as-prepared samples. (a) The response of Co_3_O_4_ HP and Pd/Co_3_O_4_ HP to different temperature at a concentration of 50 ppm. (b) Response curves of porous Co_3_O_4_ and Pd/Co_3_O_4_ HP *versus* time. (c) Response and recovery time of samples to 100 ppm ethanol at 150 °C. (d) Selectivity of Pd/Co_3_O_4_ HP sensor to ethanol, acetone, toluene, ammonia and CO at a concentration of 50 ppm.

The sensing responses of porous Pd/Co_3_O_4_ HP and pure porous Co_3_O_4_ HP for ethanol vapor were investigated. According to the sensitivity curves at different operating temperatures (Fig. 2a) and the requirement for lower working temperature, the optimum temperature of 150 °C was proposed. Fig. 2b shows the dynamic transient response of Pd/Co_3_O_4_ HP and pure Co_3_O_4_ sensors in different atmospheres at 150 °C, respectively. The response of Pd/Co_3_O_4_ HP varied from 3.8 to 24.1 when the concentration of ethanol increased from 10 ppm to 200 ppm. The response and recovery curve of Pd/Co_3_O_4_ HP and porous Co_3_O_4_ sensor upon exposure to 100 ppm ethanol vapor are exhibited in Fig. 2c, highlighting the shorter response times (12 s) and recovery times (25 s) of Pd/Co_3_O_4_ HP. To explore the selectivity of Pd/Co_3_O_4_ HP sensor, different gases were pumped to the chamber at 150 °C (Fig. 2d). Pd/Co_3_O_4_ HP exhibited the highest sensitivity (∼14.8) for 50 ppm ethanol, which can be related to kinetics and thermodynamics of gas adsorption–desorption. To explore the stability of the prepared sensor, the response transient cycles and long-term stability tests were carried out as showed in Fig. S2.† Pd/Co_3_O_4_ HP sensor to 100 ppm of ethanol at 150 °C showed a typical p-type sensing behavior. The resistance of sensor increased upon exposure to ethanol and returned to the original value in air in a repeatable way. The response of Pd/Co_3_O_4_ HP sensor has been examined for eight weeks as shown in Fig. S2b.† The sensor exhibits a nearly consistent response, which ensures the real application of Pd/Co_3_O_4_ HP sensor.

### Mechanism

Spillover effect involves the dynamic migration of surface adsorbed species between different surface sites and the dynamic changes in surface state. NAPXPS measurements were carried out to investigate dynamic changes in surface state of Pd/Co_3_O_4_ HP sensor. Various atmospheres (UHV, O_2_, and the mixture of O_2_/ethanol) were pumped into the testing chamber to simulate the working condition of Pd/Co_3_O_4_ HP sensor. The temperature was set at 150 °C, which is the optimal working temperature. The kinetic energy of 200 eV was selected in order to obtain sensitive surface information of the same atomic-layer thickness. Specifically, the XPS spectrum of O 1s was collected at the incident light energy of 730 eV and that of Pd 3d spectrum was 530 eV.

**Fig. 3:**
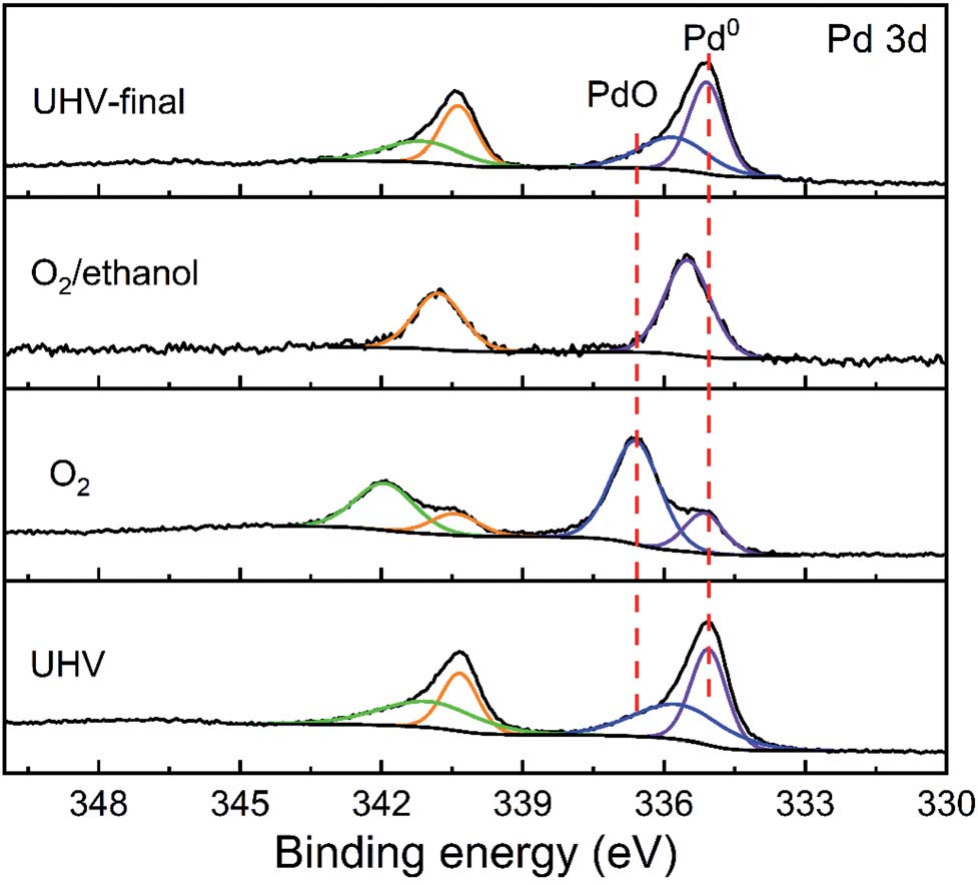
Pd 3d XPS spectrum taken on Pd/Co_3_O_4_ HP sensor exposed to different atmospheres at 150 °C.

Fig. 3 shows the XPS spectra of Pd 3d obtained under different atmosphere conditions. The peak around 335.4 eV is considered as metallic Pd,^20^ while the fitting peak near 336.9 eV is usually considered as PdO.^21^ Under UHV conditions, it is found that most of Pd on the surface is metallic. A small part of Pd 3d spectra moves slightly to the high binding energy direction due to the Schottky interaction between Pd and oxides.^22^ When oxygen was introduced, the surface Pd was oxidized rapidly and the proportion of oxidized Pd increased to 64%. Upon the further exposure of ethanol, the oxidized Pd was reduced. It is worth noting that the Pd 3d_5/2_ characteristic peak position was higher than that of metallic Pd, indicating the interaction between Pd and ethanol vapor. Finally, when the gas was extracted, Pd on the material surface returned to the initial state.

**Fig. 4:**
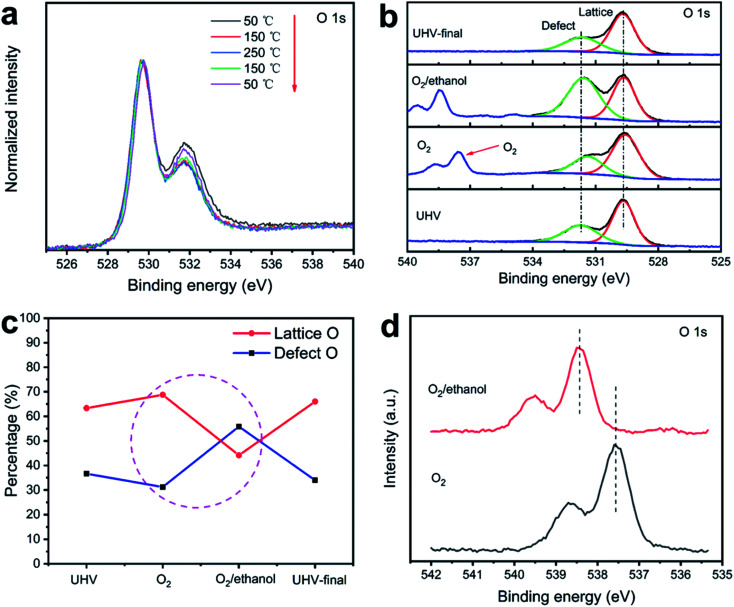
(a) O 1s spectra of Pd/Co_3_O_4_ HP at different temperatures under ultra-high vacuum; (b) O 1s spectra under different atmospheres at 150 °C; (c) percentage curves of lattice oxygen and defect oxygen; (d) O 1s spectra of gaseous oxygen.

In order to explore the intrinsic changes of oxygen species at different temperatures, O 1s at different temperatures were collected as shown in Fig. 4. Normalized O 1s spectrum (Fig. 4a) are divided into two main components as lattice oxygen and defect oxygen. It is found that the defect oxygen content decreased with the increase of temperature. When the temperature decreased, the defect oxygen content also partially recovered, which may be due to the rearrangement of surface oxygen atoms and the conversion of adsorbed oxygen at a certain temperature.^23,24^ This phenomenon confirmed that the gas sensing response performance changed with temperature.

Fig. 4b shows O 1s spectrum in different atmospheres. For simplicity, hydroxyl is also distributed in defect oxygen. It is found that the main components in O 1s did not change significantly. The peaks existed at about 529.7 eV were considered to be the lattice oxygen of Pd/Co_3_O_4_ HP, and the peaks at the binding energy of 531.8 eV corresponded to the defect oxygen. In the mixture of O_2_ or O_2_/ethanol vapor, the peak of gaseous oxygen appeared in the spectrum. The percentages of lattice oxygen and defective oxygen are shown in Fig. 4c. It is found that once ethanol gas was introduced, the proportion of defective oxygen increased significantly, while the percentage of lattice oxygen decreased, suggesting that the surface adsorbed oxygen decreased and the surface was reduced.

By measuring the binding energy of gas molecules on the sample surface and detecting its changes, the work function of sample surface and band structure can be characterized.^25^ The changes of oxygen gaseous peaks were also observed as shown in Fig. 4d. When ethanol was introduced into the chamber, the gaseous peak of oxygen moved towards high binding energy. This result indicated the change of the energy band structure of Pd/Co_3_O_4_ HP sensor contributed to the change of resistance.

**Fig. 5:**
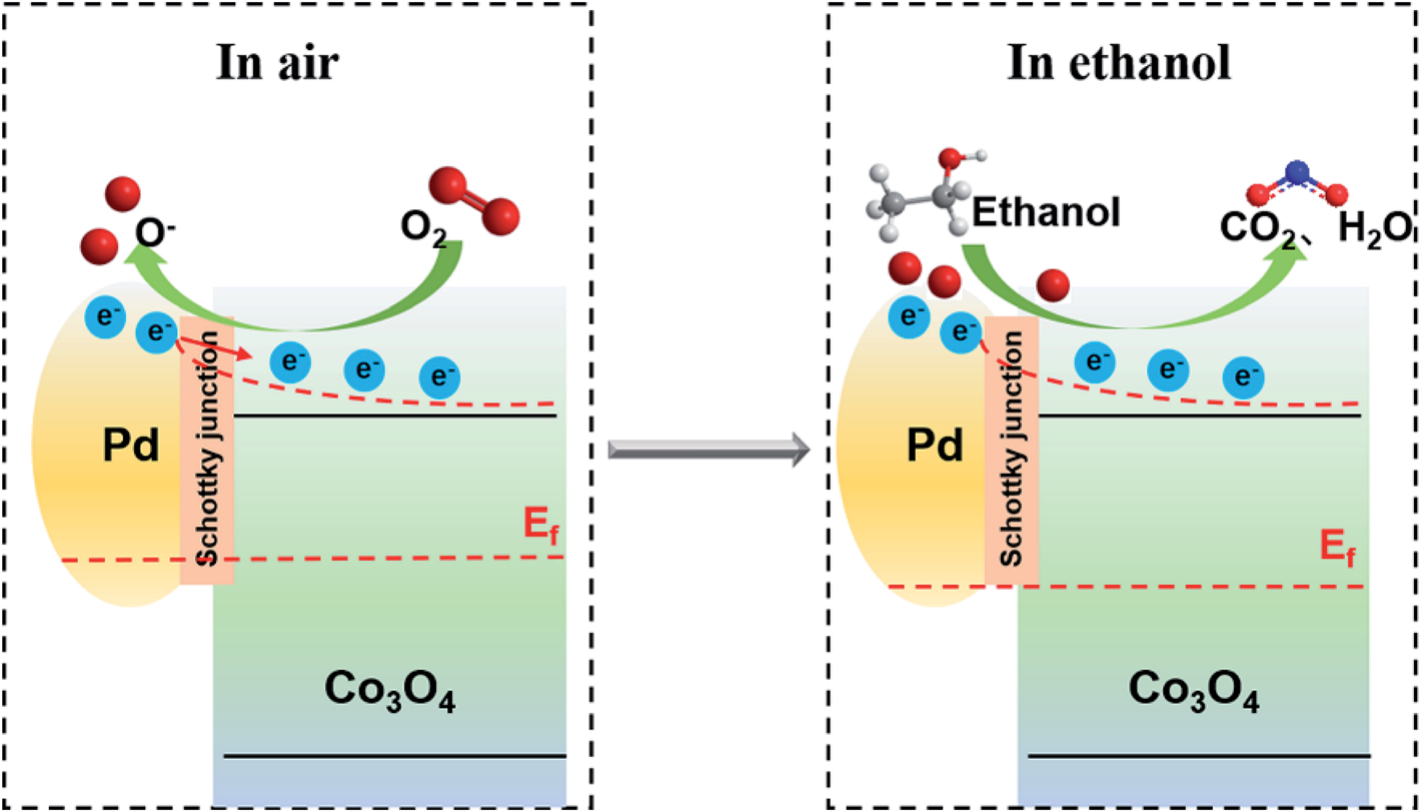
Schematic illustration of sensing mechanism in Pd/Co_3_O_4_ HP sensor.

According to above results, sensing mechanism model was proposed in Fig. 5. Pd was loaded on the oxide surface by Schottky contact while partial electrons were transferred to the oxide. In air, oxygen firstly adsorbed on the oxide surface and acquired electrons from the conduction band at the same time. Absorbed oxygen can be decomposed into reactive oxygen species and transferred to Pd surface at the operating temperature. This oxygen spillover phenomenon is proved by the oxidation of Pd 3d spectrum and no change in O 1s spectrum under O_2_ condition. Once ethanol vapor was introduced, ethanol rapidly reacted with reactive oxygen absorbed on the surface of Pd, while Pd was reduced. The electrons were released to the semiconductor, which allowed the sensor resistance to be restored. In this work, the dynamic changes in the surface state of Pd/Co_3_O_4_ HP were studied by NAPXPS, which help further explain the spillover sensing mechanism in metal–support oxide sensors.

## 4. Conclusions

In summary, Pd/Co_3_O_4_ HP was fabricated by one-step pyrolysis of Pd-MOF precursor. Due to the combination of unique structure and catalytic sensitization of Pd, Pd/Co_3_O_4_ HP sensor achieve 1.6 times higher sensitivity than that of Co_3_O_4_ HP along with fast response (12 s) and recovery speed (25 s) for 100 ppm ethanol vapor at an optimal operating temperature of 150 °C. NAPXPS was used to monitor the dynamic changes in the surface state of Pd/Co_3_O_4_ HP. It was presented that the phenomenons of oxidation and reduction of Pd in ethanol sensing process were attributed to spillover effect of oxygen and ethanol. This work provides experimental guidance for gas sensors to effectively enhance the ability of gas detection by Pd reined spillover effect, which opens up a unique approach to investigate sensing mechanism of metal–support oxides as high-performance gas sensors.

## Conflicts of interest

All authors declare that there are no conflicts of interest.

## Supplementary Material

RA-012-D1RA09352E-s001
