# Supplementary material for: Highly sensitive and fast-response ethanol sensing of porous Co3O4 hollow polyhedra via palladium reined spillover effect
Source: RSC Adv. 2022 Feb 28;12(11):6725–31. doi: 10.1039/d1ra09352e (PMC8981977; doi:10.1039/d1ra09352e)
Supplement: RA-012-D1RA09352E-s001 [file RA-012-D1RA09352E-s001.pdf]

Electronic Supplementary Material (ESI) for RSC Advances.
This journal is © The Royal Society of Chemistry 2022

Highly sensitive and fast-response ethanol sensing of porous Co_3O_4 hollow polyhedra via palladium reined spillover effect

Guotao Yuan[1], Yihong Zhong[1], Yufeng Chen[1], Qiqi Zhuo[2,] & Xuhui Sun[1,*]*

[1]Institute of Functional Nano and Soft Materials Laboratory (FUNSOM), Soochow University, Suzhou 215123, China

[2]College of Material Science & Engineering, Jiangsu University of Science and Technology, Zhenjiang, China

[*] Corresponding authors: Email: zqq88263268@126.com (Q. Zhuo); xhsun@suda.edu.cn (X. Sun).

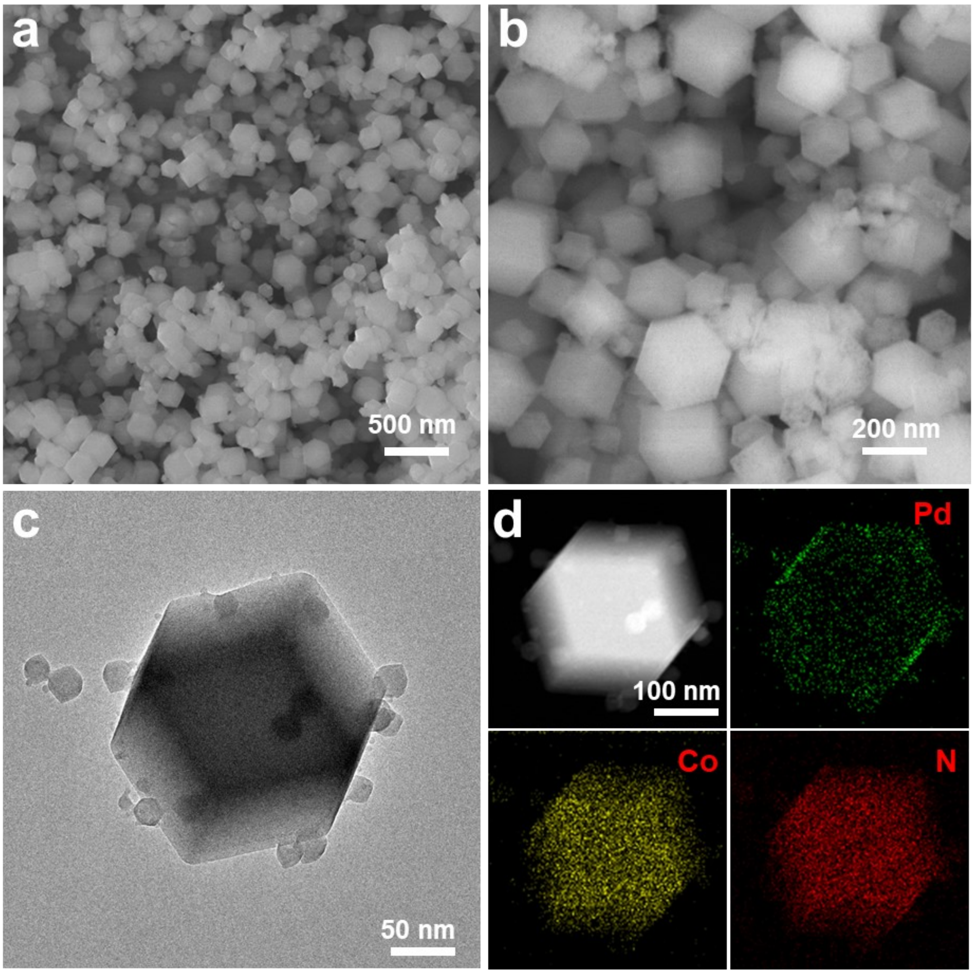

Figure S1 (a) and (b) the SEM images of Pd-ZIF-67 with different magnifications; (c) TEM image and (d) corresponding EDX mappings of Pd, Co, and N elements

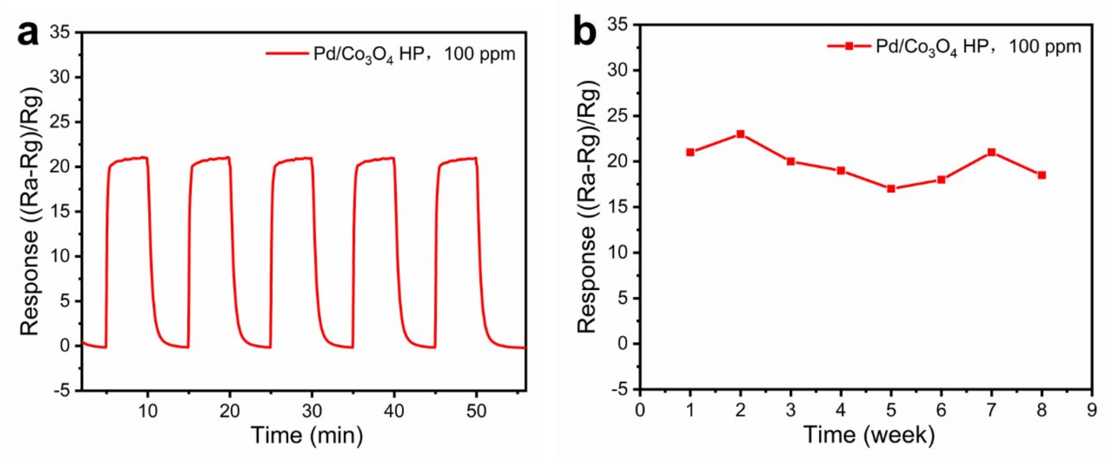

Figure S2 The sensing stability tests. (a) the response transient cycles and (b) long-term stability testing curve of Pd/Co_3O_4 HP sensor to 100 ppm of ethanol at 150 °C.

Table S1 Gas sensing performances based on various nanostructures of Co_3O_4 composites in literatures and the present work.

**Target gas**	**Materials**	**Morphology**	**Temp. (°C)**	**Con. (ppm)**	**Res. (Rg/Ra)**	**Res./Rec. times (s)**	**Ref**
Ethanol	Co_3O_4	nanosheet	300	100	57.7	66/10	[1]
	Cr/ Co_3O_4	nanosheet	300		24.6	1/7	[2]
	Co_3O_4/CF	flower	320		4.2	44/31	[3]
	Co_3O_4/NCF	nanosheet	100		10.4	45/140	[4]
	ZnO/Co_3O_4	microsphere	275		~60	5.6/29	[5]
	Pd/ Co_3O_4	Hollow polyhedral	150		21	12/25	This work

References

1. Choi, K.-I.; Kim, H.-R.; Kim, K.-M.; Liu, D.; Cao, G.; Lee, J.-H. *Sensors and Actuators B: Chemical* **2010,** 146, 183-189.

2. Zhang, P.; Wang, J.; Lv, X.; Zhang, H.; Sun, X. *Nanotechnology* **2015,** 26, 275501.

3. Li, L.; Liu, M.; He, S.; Chen, W. *Anal. Chem.* **2014,** 86, 7996-8002.

4. Li, L.; Zhang, C.; Zhang, R.; Gao, X.; He, S.; Liu, M.; Li, X.; Chen, W. *Sensors and Actuators B: Chemical* **2017,** 244, 664-672.

5. Qu, F.; Thomas, T.; Zhang, B.; Zhou, X.; Zhang, S.; Ruan, S.; Yang, M., *Sensors and Actuators B: Chemical* **2018**, 273, 1202-1210.
